# Editorial: Lipid Signaling in T Cell Development and Function

**DOI:** 10.3389/fimmu.2015.00410

**Published:** 2015-08-10

**Authors:** Karsten Sauer, Klaus Okkenhaug

**Affiliations:** ^1^Department of Immunology and Microbial Science, The Scripps Research Institute, La Jolla, CA, USA; ^2^Department of Cell and Molecular Biology, The Scripps Research Institute, La Jolla, CA, USA; ^3^Laboratory of Lymphocyte Signalling and Development, Babraham Institute, Cambridge, UK

**Keywords:** lipid, PI3K, T cell, inositol, eicosanoid, vitamin D_3_, adipokine, TNF

Best known as energy storing molecules and core-components of cellular membranes, lipids are also important regulators of cell signaling. They can compartmentalize signaling by organizing membrane-topology into specialized sub-domains in the plasma membrane or into intracellular microvesicles (1, 2). Covalent lipid-modification of proteins can direct their localization to particular membrane compartments. Non-covalent membrane lipid interactions control the mechanics of T cell receptor (TCR) signal transduction (3, 4). Membrane lipids can also act as second-messengers, as exemplified by the phosphoinositide-3-kinase (PI3K) lipid-product phosphatidylinositol(3,4,5)trisphosphate (PIP_3_) in lymphocytes, the topic of several reviews here, and of a recent dedicated Research Topic in *Frontiers in Immunology* (5). Other lipids can also act as intracellular or extracellular messengers, or govern cell function at the level of metabolism. Not surprisingly, lipid producing and metabolizing enzymes and lipid downstream-effectors have gained considerable attention as potential therapeutic targets for immune disorders, blood cancer, and even aging (5, 6) (and this Research Topic), and certain lipids are being used as therapeutics (7).

The 11 reviews in this Research Topic highlight some of the most important, or most recently discovered lipid functions in T cells. So and Croft review evidence that besides the TCR and costimulatory CD28, members of the Tumor-Necrosis-Factor (TNFR) superfamily contribute to sustained PI3K-pathway activation in T cells and beyond (8). Based on their studies of the TNFR OX40, the authors suggest a model where ligand-induced TNFR oligomerization concentrates PI3K and Akt close to TCR/CD28 signalosomes. This may contribute to the well-established TNFR-requirements for T cell clonal expansion, survival, and memory. The authors also review evidence for TNFR-mediated PI3K control in other cells and discuss important open questions, such as which precise molecular interactions link TNFRs to PI3K/Akt. Wang and colleagues review the components and mechanisms of PI3K-signaling in lymphocytes (9). They discuss how PI3Ks are activated to produce PIP_3_, the mechanisms limiting PIP_3_ production, and the role of PIP_3_ removal by lipid-phosphatases. Next, the authors discuss how PIP_3_ specifically binds to effector proteins such as Akt and Tec-kinases via their PH domains to control lymphocyte biology. They also discuss a less well-appreciated mechanism of how soluble inositol-phosphates can control PI3K-signaling by acting as PIP_3_-analogs. An increasing number of studies suggest that this non-canonical way of controlling PI3K-function has broad importance in hematopoiesis (9–11). The authors conclude by reviewing how protein-ligands of PIP_3_-binding domains provide yet another level of control as exemplified by their recent work on calmodulin–PH domain interactions. A complementing review by Srivastava and colleagues focuses on PIP_3_ metabolizing lipid-phosphatases in T cells (12). The paramount tumor suppressor function of PTEN, critical functions in effector and regulatory T cells, and recent efforts to target them pharmacologically underscore the importance of PIP_3_ removal by lipid-phosphatases. Among them, PTEN reverses the PI3K-reaction, whereas SHIP1/2 also control signaling by producing PI(3,4)P_2_, which recruits and controls effector proteins additional to Akt and Tec-family kinases.

But PIP_3_ and its immediate derivatives are not the only important phosphoinositides in immune cells. Nunès and Guittard discuss recent evidence for functions of PI5P in TCR signaling (13). They introduce the enzymes governing PI5P metabolism, review PI5P-binding domains in effector proteins such as Dok, and discuss potential PI5P functions in T cells. While inositol-phosphates are produced through hydrolysis of the phosphodiester-bond between the glycerol and inositol-phosphate moieties of phosphatidylinositols, phosphatidylinositol-deacylation by phospholipase A generates glycerophosphoinositols. Containing both glycerol- and phosphoinositol-moieties, glycerophosphoinositols are soluble and may have intracellular signaling roles. They can also be excreted to potentially exert paracrine functions. Patrussi et al. discuss metabolism and potential immunomodulatory roles for these little-studied messengers (14). In particular, glycerophosphoinositol(4)phosphate can augment TCR and CXCR4 chemokine receptor signaling in T cells. Unknown cellular receptors for, and physiological relevance of glycerophosphoinositols indicate exciting research opportunities in this young field.

Other phosphoinositide derivatives with important functions in T cells are the PI3K-substrate phosphatidylinositol(4,5)bisphosphate (PIP_2_), diacylglycerol (DAG) and phosphatidic acid (PA). Jun and colleagues review how these lipids orchestrate intricate interactions of Ras guanine-nucleotide-exchange-factors, Ca^2+^, adaptor- and effector proteins to control both kinetics and topology of Ras-activation in T cells (15). The authors discuss the roles of allosteric and feedback mechanisms, Ras-acylation, and complex interactions between the Ras- and PI3K-pathways. A complementing review by Krishna and Zhong discusses how diacylglycerol-kinases (DGK) phosphorylate DAG into PA to control T cell development and function, in particular to maintain self-tolerance (16). The authors review roles for known DAG-effectors and little-understood PA-effectors in T cells, potential contributions of DAG or PA metabolism, and DGK functions in other immune cells. Improved antiviral and antitumor activities of DGK-deficient T cells might indicate potential translational relevance, but as the authors point out, more studies are needed.

Moving beyond phosphoinositides, Nicolaou and colleagues (7) and Lone and colleagues (17) review the diverse functions of polyunsaturated fatty acid (PUFA)-derivatives in T cells. PUFA-lipids can modulate membrane-associated signalosomes by altering membrane composition, or act as precursors of secreted signaling-lipids. Both reviews first discuss biosynthesis and metabolism of eicosanoids, including prostanoids, leukotrienes, fatty acid epoxides, and endocannabinoids, and then review their diverse and often complex functions in T cell biology and disease. They further discuss the therapeutic potential of PUFA, a particularly interesting topic given the exploration of dietary PUFA as antiinflammatory agents, and the interest in targeting the prostanoid PGE_2_ to improve immunotherapies for cancer or infections (7, 18).

Another lipid-derived messenger with important immune-regulatory functions is the cholesterol-derivative vitamin D_3_. Kongsbak and colleagues review how its binding to the transcription-factor vitamin-D-receptor (VDR) controls T cell development, differentiation, and function (19). They describe the mechanisms controlling VDR expression and activity, and discuss roles for VDR downregulation in promoting autoimmune diseases or in dampening innate immunity during certain infections. Autoimmune disease-reversal by VDR-agonist/antibiotic combinations suggests translational relevance and possible contributions to the long-known but ill-understood links between microbial infections and the etiology of autoimmmunity. Available as nutrients, vitamin D_3_, the lipid-related vitamin A, and short-chain fatty acids (20) all are potential lead-agents for therapeutic immunomodulation.

Finally, Procaccini and colleagues discusses how adipokine-hormones – produced by fat tissue and best known to influence energy-homeostasis and neuroendocrine function – may link metabolism with immunity (21). This area has recently gained increasing attention because of reported links between obesity, chronic inflammation, and various diseases, including cancer. The authors introduce the cellular and molecular components linking fat tissue and immune system and then review the often controversial, potential immunoregulatory roles of leptins, adiponectins, and other adipokines.

Altogether, the reviews in this Research Topic highlight how recent progress has profoundly altered and expanded our understanding of lipid functions in T cell biology but also raised many interesting questions. Rather than merely acting as membrane components and energy stores, lipids have emerged as important and multifaceted signaling molecules both inside and outside of T cells. We believe that this *Frontiers in Immunology* Research Topic provides its readers with a broad and stimulating basis to follow these important developments. In this sense, we thank all the authors for their outstanding contributions.

## Conflict of Interest Statement

The authors declare that the research was conducted in the absence of any commercial or financial relationships that could be construed as a potential conflict of interest.
